# Author Correction: Dysfunctional ERG signaling drives pulmonary vascular aging and persistent fibrosis

**DOI:** 10.1038/s41467-022-33197-w

**Published:** 2022-09-28

**Authors:** Nunzia Caporarello, Jisu Lee, Tho X. Pham, Dakota L. Jones, Jiazhen Guan, Patrick A. Link, Jeffrey A. Meridew, Grace Marden, Takashi Yamashita, Collin A. Osborne, Aditya V. Bhagwate, Steven K. Huang, Roberto F. Nicosia, Daniel J. Tschumperlin, Maria Trojanowska, Giovanni Ligresti

**Affiliations:** 1 Department of Physiology & Biomedical Engineering, Mayo Clinic, Rochester, MN USA; 2 Department of Medicine, Boston University School of Medicine, Boston, MA USA; 3 Department of Medicine, Perelman School of Medicine, University of Pennsylvania, Philadelphia, PA USA; 4 Department of Biomedical Statistics and Informatics, Mayo Clinic, Rochester, MN USA; 5 Department of Internal Medicine, University of Michigan Medical School, Ann Arbor, MI USA; 6 Department of Laboratory Medicine and Pathology, University of Washington, Seattle, WA USA

**Keywords:** Respiratory tract diseases, Chromatin

Correction to: *Nature Communications* 10.1038/s41467-022-31890-4, published online 25 July 2022

The original version of this article contained errors in Figure 3d–f and Figure 4f–h, in which the scale bars were white, instead of colored. These errors have been corrected in the PDF and HTML version of the article.

