# Supplementary material for: Dysfunctional ERG signaling drives pulmonary vascular aging and persistent fibrosis
Source: Nat Commun. 2022 Jul 25;13:4170. doi: 10.1038/s41467-022-31890-4 (PMC9314350; doi:10.1038/s41467-022-31890-4)

Dysfunctional ERG signaling drives pulmonary vascular aging and persistent fibrosis

Nunzia Caporarello[1#], Jisu Lee[2#], Tho X. Pham[2], Dakota L. Jones[3], Jiazhen Guan[2], Patrick A. Link[1], Jeffrey A. Meridew[1], Grace Marden[2], Takashi Yamashita[2], Collin A. Osborne[4], Aditya V. Bhagwate[4], Steven K. Huang[5], Roberto F. Nicosia[6], Daniel J. Tschumperlin[1], Maria Trojanowska[2], Giovanni Ligresti[2]

[#] These authors contributed equally to this work.

SUPPLEMENTARY INFORMATION

Figure S1

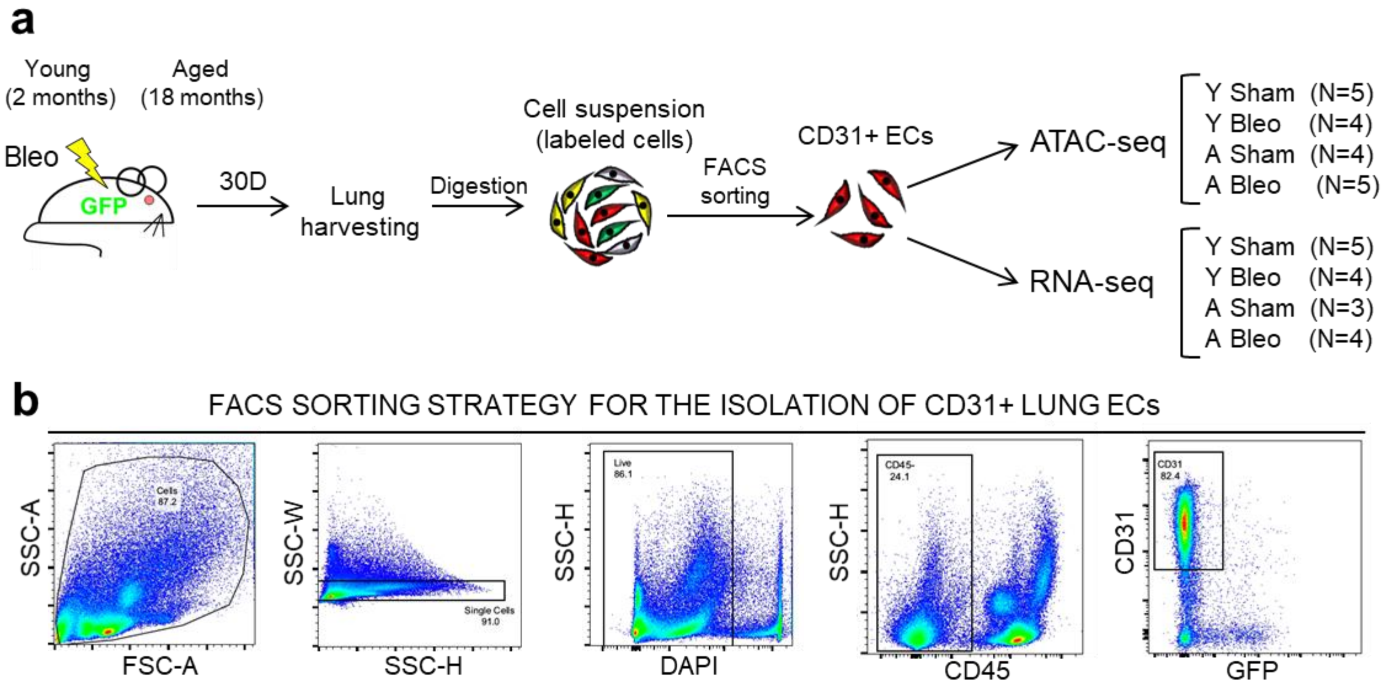

Supplementary figure 1. Isolation of lung ECs for comparative ATAC-seq and RNA-seq analysis. **a)** Col1a1 GFP reporter mice (young and aged) were exposed to a single dose of bleomycin and sacrificed at day 30. Lungs were harvested and prepared for FACS sorting. Freshly sorted lung ECs were subjected to comparative ATAC-seq and RNA-seq analysis. **b).** Schematic showing the strategy used to isolate lung ECs. Single cell suspensions were depleted from debris, doublet and dead cells followed by CD45, CD326 and GFP depletion and by CD31+ selection.

Figure S2

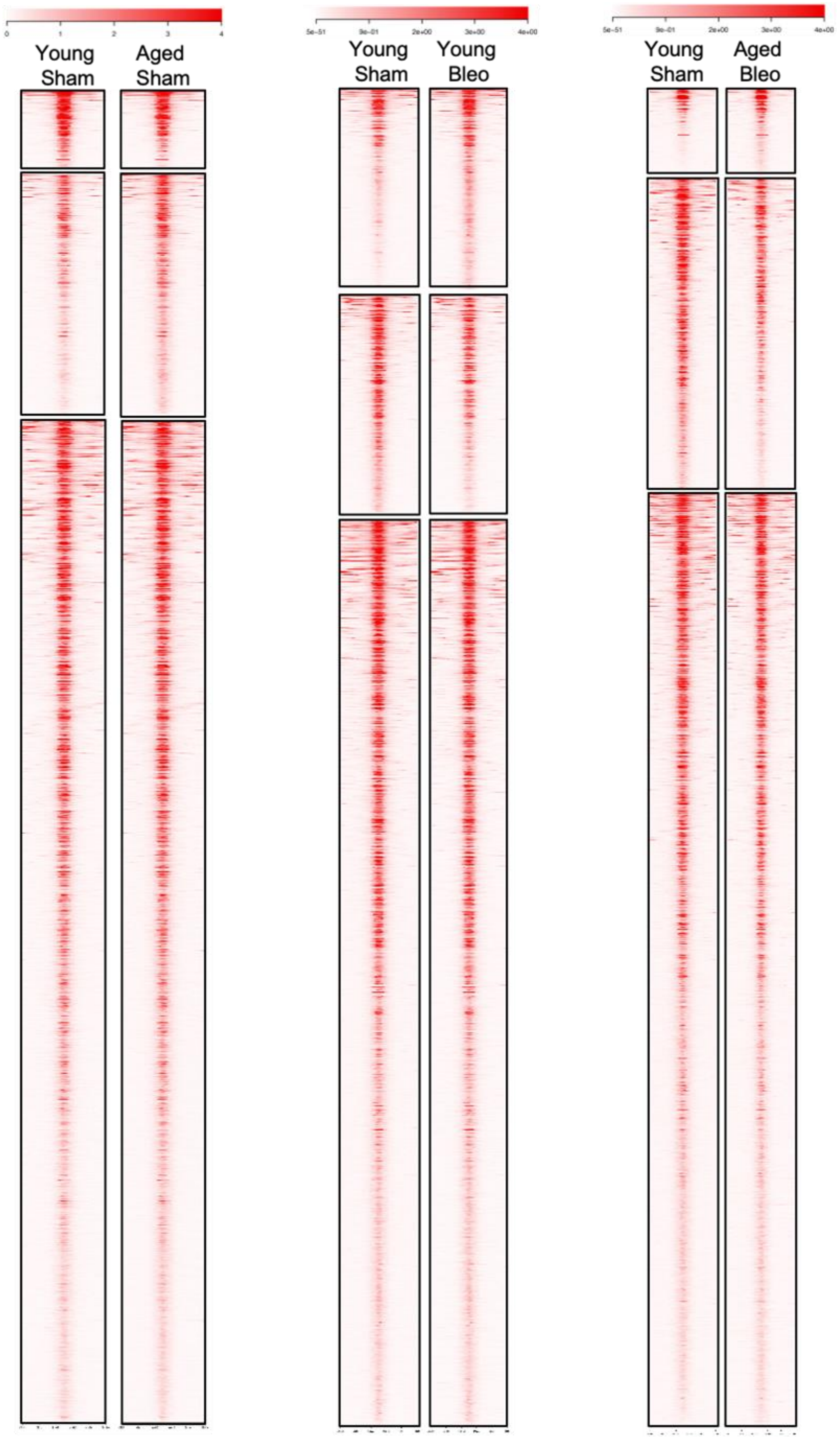
0
1
2
3
4
Young Sham
Aged Sham
5e-51
9e-01
2e+00
3e+00
4e+00
Young Sham
Young Bleo
5e-51
9e-01
2e+00
3e+00
4e+00
Young Sham
Aged Bleo

Supplementary figure 2. Lung endothelial chromatin accessibility is reduced with aging

Heatmaps showing the relative numbers of peaks in regions exhibiting reduced signal, increased signal or unchanged signal for each of the following comparisons: young sham *vs* aged sham **(a)**, young sham *vs* young bleo **(b)** and young sham *vs* aged bleo **(c)**.

Figure S3

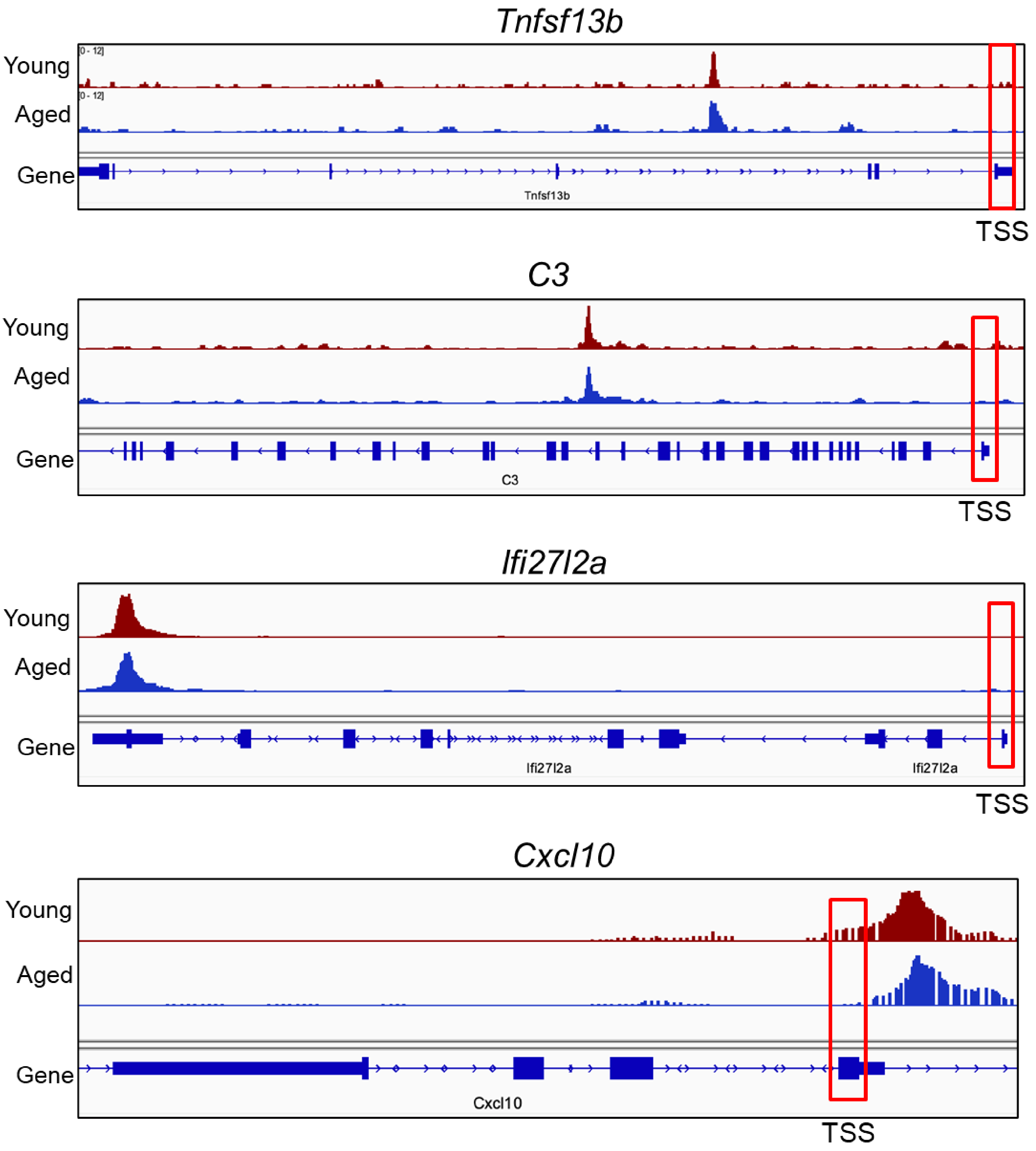
Tnfsf13b
Young
Aged
Gene
[0 - 12]
[0 - 12]
Tnfsf13b
TSS
C3
Young
Aged
Gene
C3
TSS
Ifi27l2a
Young
Aged
Gene
Ifi27l2a
Ifi27l2a
TSS
Cxcl10
Young
Aged
Gene
Cxcl10
TSS

Supplementary figure 3. Chromatin peak distribution in genes exhibiting increased expression in aged lung ECs.

Genomic snapshots depicting the absence of peaks at the promoters of representative inflammatory genes in lung ECs from young and aged mice in the absence of injury or a reduced peak in lung ECs from aged mice compared to young ones (*Cxcl10*). This pattern does not correlate with the gene expression of these genes, which was found to be elevated in uninjured aged lung ECs compared to young ones.

Figure S4

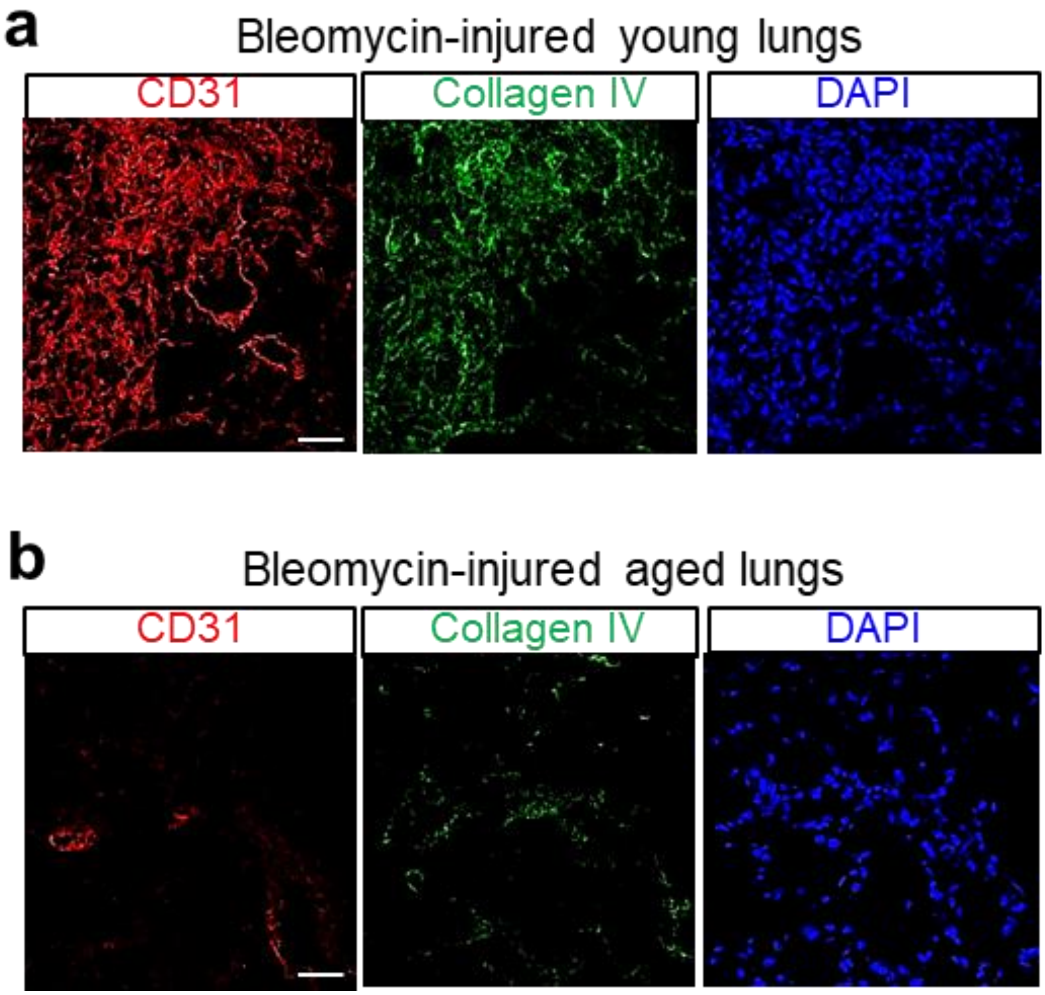

Supplementary figure 4. Basement membrane proteins are reduced in aged lungs after bleomycin injury.

a, b) Representative immunofluorescence images of injured young and aged lungs stained with CD31 antibody (red), Collagen IV antibody (green) and DAPI (blue) show reduced Collagen IV secretion in areas of active fibrosis and limited vascularization in aged animals (Young bleomycin n=4; Aged bleomycin n=5). Scale bar: 100 μM.

Figure S5

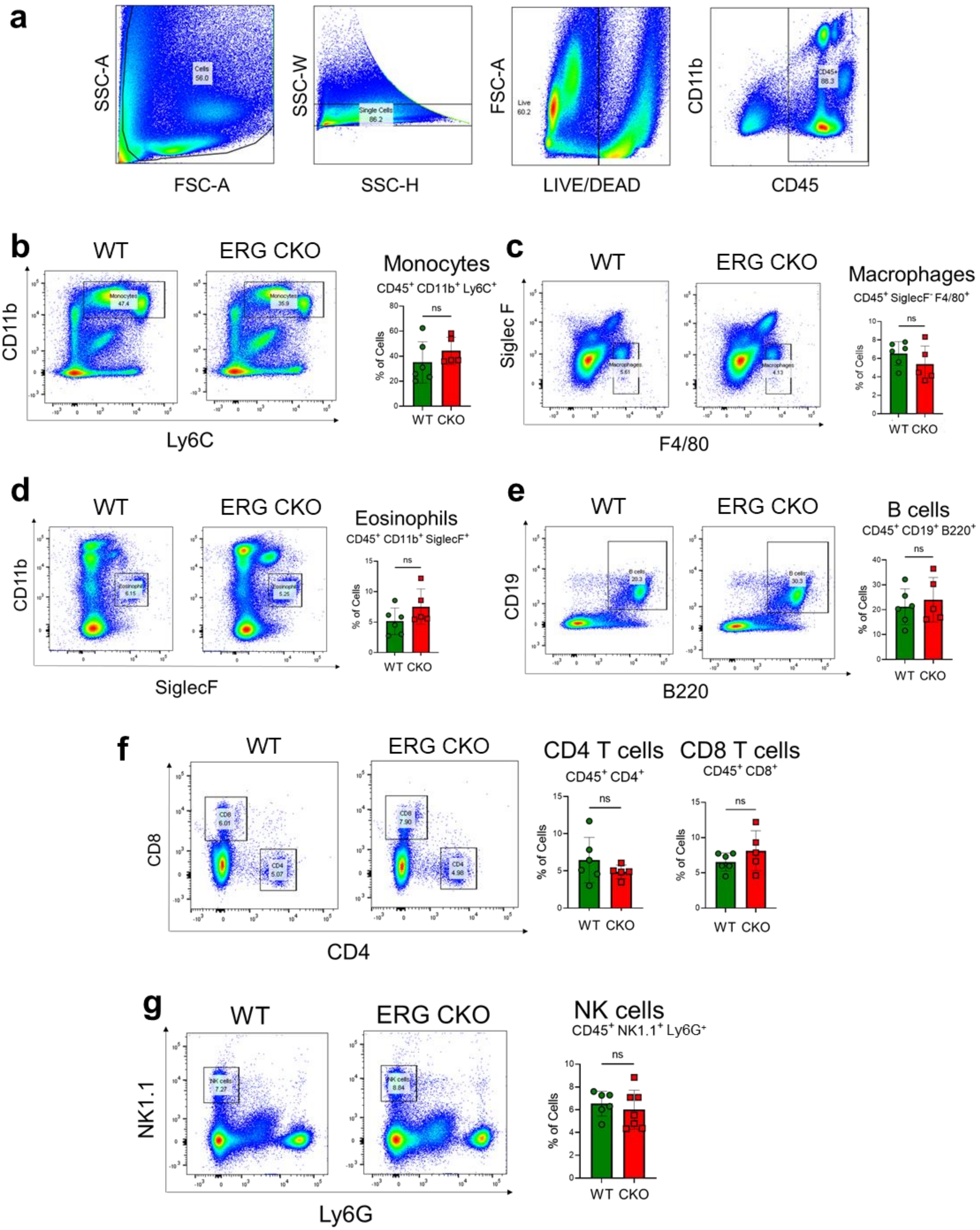
a
SSC-A
FSC-A
Cells
56.0
SSC-W
SSC-H
Single Cells
86.2
FSC-A
LIVE/DEAD
Live
60.2
CD11b
CD45
CD45+
88.3
b
WT
ERG CKO
CD11b
Ly6C
Monocytes
47.4
35.9
Monocytes
CD45+ CD11b+ Ly6C+
ns
% of Cells
WT CKO
c
WT
ERG CKO
Siglec F
F4/80
Macrophages
5.61
4.13
Macrophages
CD45+ SiglecF- F4/80+
ns
% of Cells
WT CKO
d
WT
ERG CKO
CD11b
SiglecF
6.15
5.25
Eosinophils
CD45+ CD11b+ SiglecF+
ns
% of Cells
WT CKO
e
WT
ERG CKO
CD19
B220
B cells
20.3
30.3
B cells
CD45+ CD19+ B220+
ns
% of Cells
WT CKO
f
WT
ERG CKO
CD8
CD4
CD8
6.01
CD4
5.07
CD8
7.90
CD4
4.98
CD4 T cells
CD45+ CD4+
ns
% of Cells
WT CKO
CD8 T cells
CD45+ CD8+
ns
% of Cells
WT CKO
g
WT
ERG CKO
NK1.1
Ly6G
NK cells
7.27
NK cells
8.84
NK cells
CD45+ NK1.1+ Ly6G+
ns
% of Cells
WT CKO

Supplementary figure 5. Characterization of immune cell subsets in the lungs of WT and ERG CKO mice

FACS strategy used to identify and analyze multiple immune cell populations in whole lungs of WT and ERG CKO mice. Single cell suspensions were depleted from debris, doublet and dead cells. The immune cells were then characterized based on specific membrane markers as following: Monocytes ($CD45^+$, $CD11b^+$, $Ly6C^+$, WT n=6, CKO n=5), Macrophages ($CD45^+$, $SiglecF^+$, $F4/80^+$, WT n=6, CKO n=5), Eosinophils ($CD45^+$, $CD11b^+$, $SiglecF^+$, WT n=6, CKO n=5), B cells ($CD45^+$, $CD19^+$, $B220^+$, WT n=6, CKO n=5), $CD4^+$ T cells ($CD45^+$, $CD4^+$, WT n=6, CKO n=5), $CD8^+$ T cells ($CD45^+$, $CD8^+$, WT n=6, CKO n=5), NK cells ($CD45^+$, $NK1.1^+$, $Ly6G^+$, WT n=6, CKO n=7). No appreciable differences were observed between WT ans ERG CKO animals. All values are summarized as mean and SD. Source data are provided as a Source Data file.

Figure S6

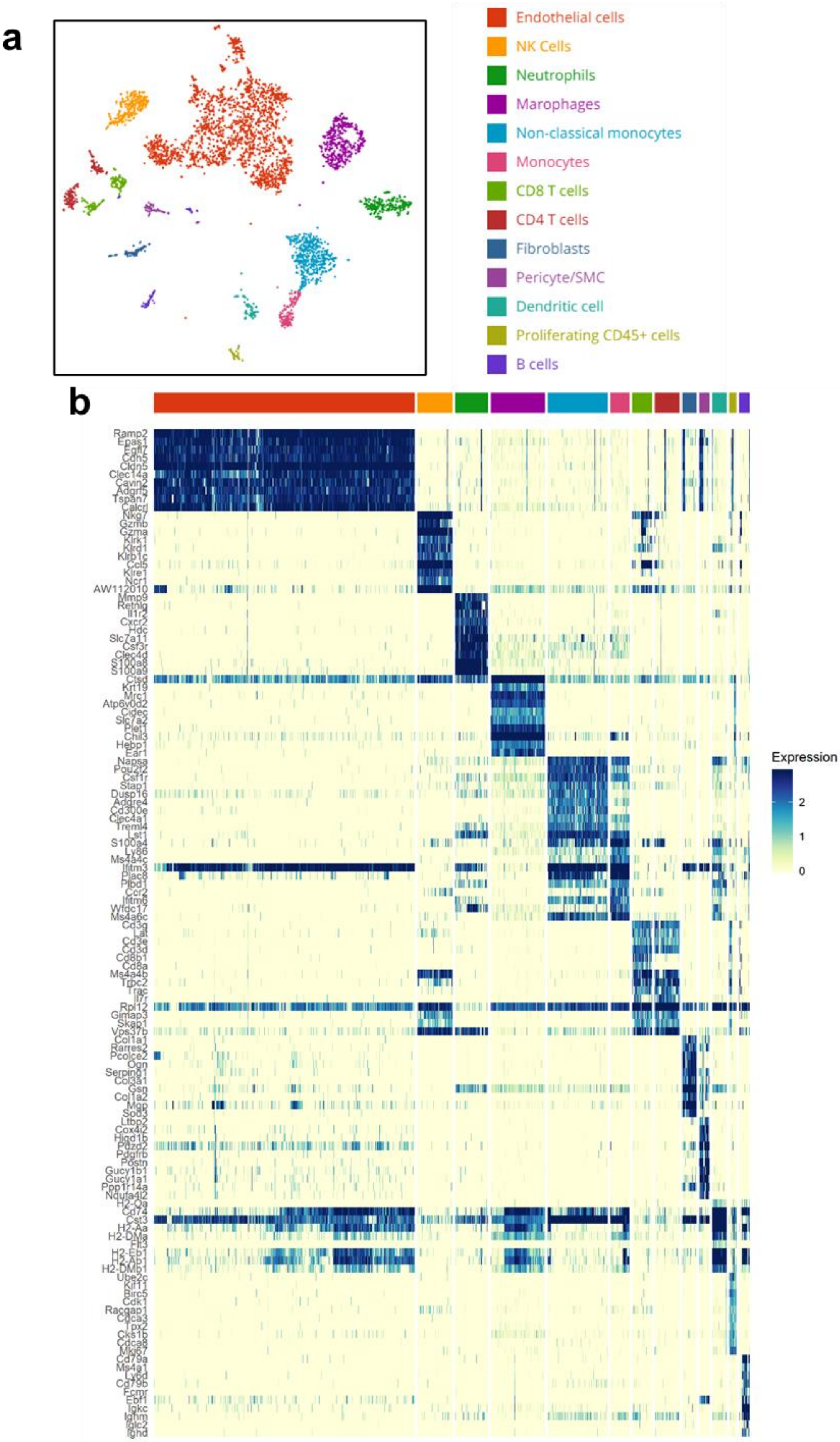
a
Endothelial cells
NK Cells
Neutrophils
Marophages
Non-classical monocytes
Monocytes
CD8 T cells
CD4 T cells
Fibroblasts
Pericyte/SMC
Dendritic cell
Proliferating CD45+ cells
B cells
b
Expression
2
1
0

Supplementary figure 6. Results of scRNA-seq profiles of mouse lung endothelial cells.

a) *t*-SNE plot of 13 distinct cell populations from scRNA-seq data of WT and ERG CKO lungs. **b).**

Heatmap of marker gene expression that distinguishes cell populations of WT and ERG CKO lungs.

Figure S7

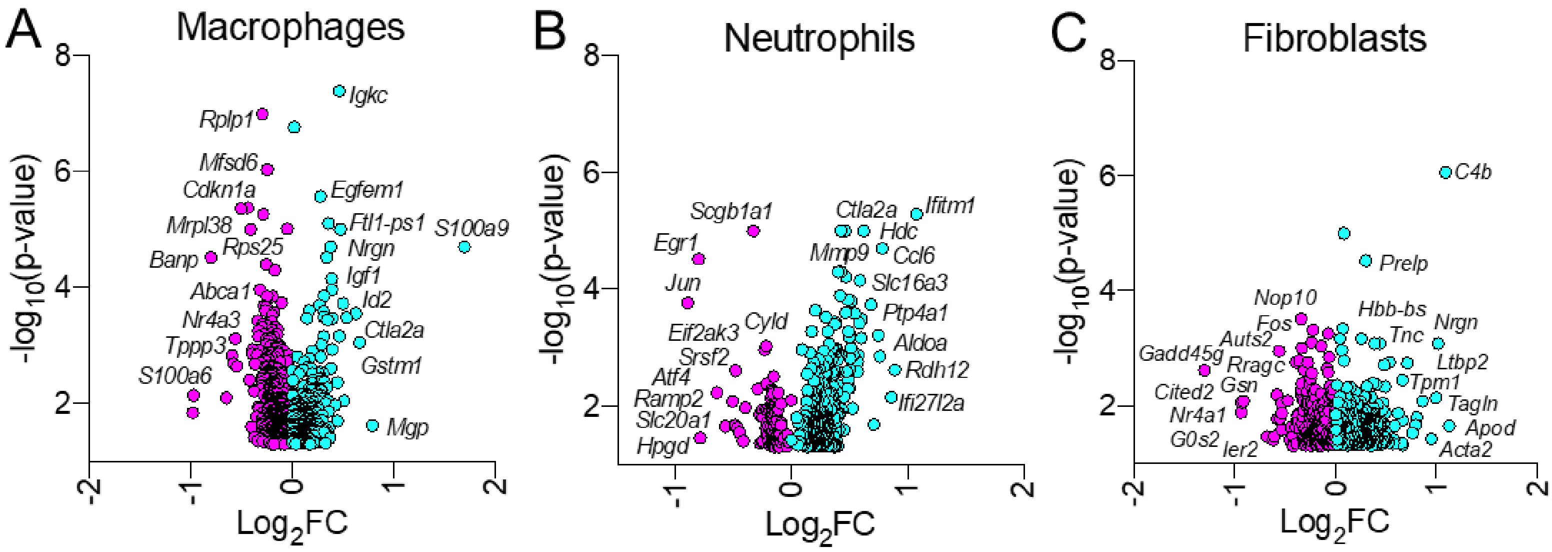

Supplementary figure 7. Differential expressed genes in non-vascular lung cells from ERG CKO mice compared to WT ones.

a-c) Volcano plots showing differentially expressed genes in macrophages, neutrophils, and fibroblasts from ERG CKO mice compared to WT mice. Differential expression of single cell data was performed within BBrowser using the Venice algorithm. Pink dots represent downregulated genes in ERG CKO animals compared to WT ones, whereas aqua dots represent genes upregulated in ERG CKO animals compared to WT ones.

Figure S8

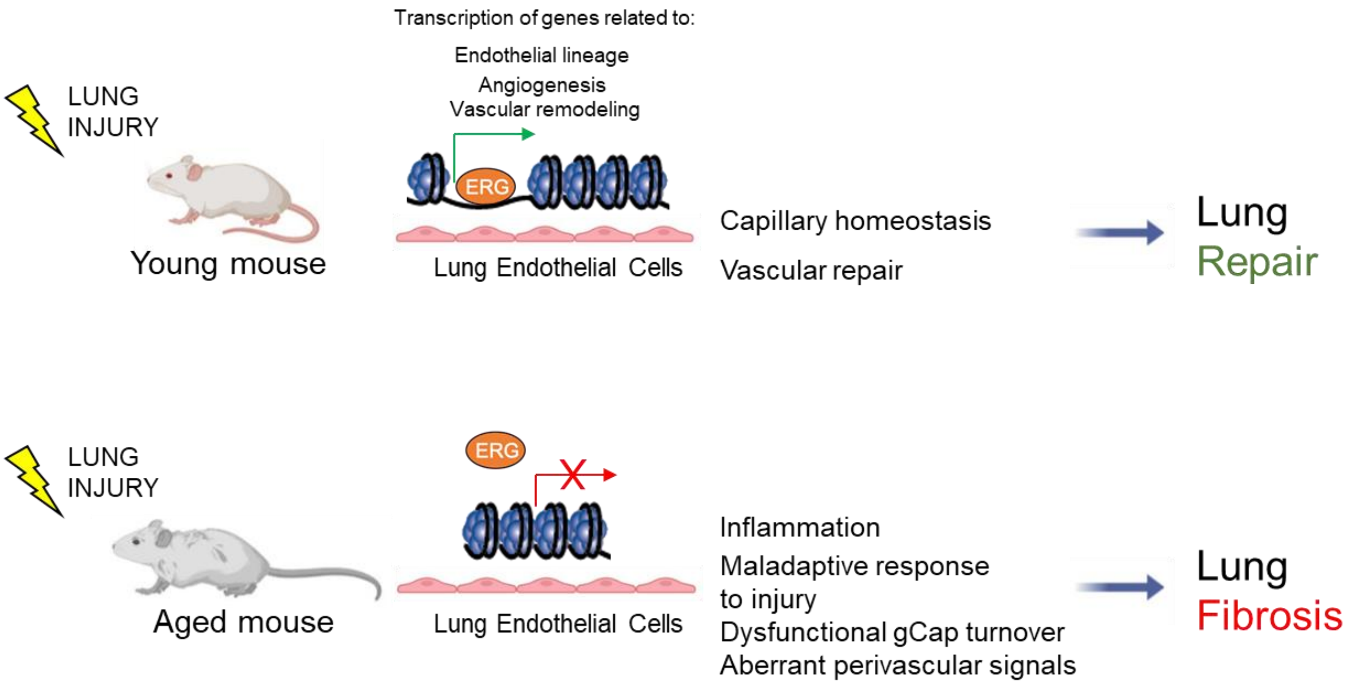

Supplementary figure 8. Schematic showing the putative mechanisms leading to dysfunctional ERG signaling in aging.

In young mice, ERG orchestrates endothelial chromatin remodeling during lung fibrosis resolution. In contrast, altered ERG/chromatin interaction leads to maladaptive transcriptional response to injury and impairs lung fibrosis resolution.

Uncropped gels – Figure 5F

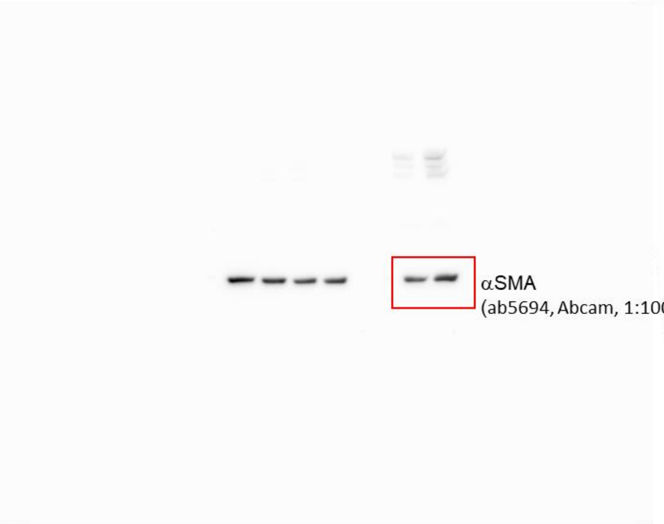

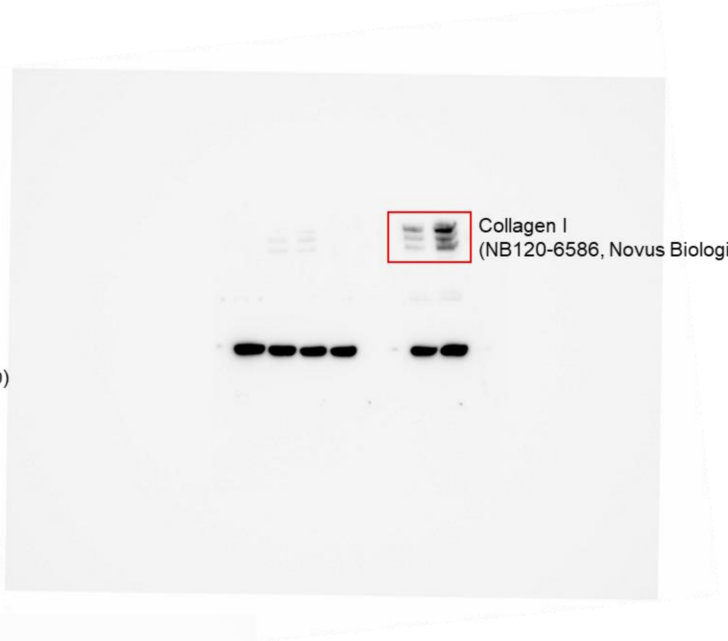

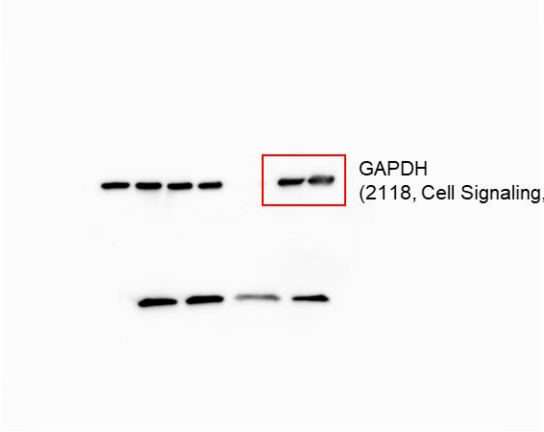

Supplement: Supplementary file 1 — Supplementary Information [file 41467_2022_31890_MOESM1_ESM.pdf]
