# Supplementary material for: Dysfunctional ERG signaling drives pulmonary vascular aging and persistent fibrosis
Source: Nat Commun. 2022 Jul 25;13:4170. doi: 10.1038/s41467-022-31890-4 (PMC9314350; doi:10.1038/s41467-022-31890-4)
Supplement: Supplementary file 2 — Reporting Summary [file 41467_2022_31890_MOESM2_ESM.pdf]

nature portfolio

Corresponding author(s): Giovanni Ligresti

Last updated by author(s): May 13, 2022

Reporting Summary

Nature Portfolio wishes to improve the reproducibility of the work that we publish. This form provides structure for consistency and transparency in reporting. For further information on Nature Portfolio policies, see our Editorial Policies and the Editorial Policy Checklist.

Statistics

For all statistical analyses, confirm that the following items are present in the figure legend, table legend, main text, or Methods section.

n/a	Confirmed	
☐	☒	The exact sample size (n) for each experimental group/condition, given as a discrete number and unit of measurement
☐	☒	A statement on whether measurements were taken from distinct samples or whether the same sample was measured repeatedly
☐	☒	The statistical test(s) used AND whether they are one- or two-sided<br>*Only common tests should be described solely by name; describe more complex techniques in the Methods section.*
☐	☒	A description of all covariates tested
☐	☒	A description of any assumptions or corrections, such as tests of normality and adjustment for multiple comparisons
☐	☒	A full description of the statistical parameters including central tendency (e.g. means) or other basic estimates (e.g. regression coefficient) AND variation (e.g. standard deviation) or associated estimates of uncertainty (e.g. confidence intervals)
☐	☒	For null hypothesis testing, the test statistic (e.g. F, t, r) with confidence intervals, effect sizes, degrees of freedom and P value noted<br>*Give P values as exact values whenever suitable.*
☒	☐	For Bayesian analysis, information on the choice of priors and Markov chain Monte Carlo settings
☒	☐	For hierarchical and complex designs, identification of the appropriate level for tests and full reporting of outcomes
☐	☒	Estimates of effect sizes (e.g. Cohen's d, Pearson's r), indicating how they were calculated

Our web collection on statistics for biologists contains articles on many of the points above.

Software and code

Policy information about availability of computer code

Data collection	The HiChIP pipeline (including MACS2 v2.0.10, BWA v0.5.9, Bedtools v2.16.2, IGV v2.8.13) was used to process all fastq files into the respective alignment, peak, and visualization files. All in house scripts are described in https://bmcbioinformatics.biomedcentral.com/articles/10.1186/1471-2105-15-280 or are publicly available through zenodo (https://doi.org/10.5281/zenodo.6543252).
Data analysis	Diffbind was used to identify differential peaks to then be annotated and have motifs identified using HOMER. Typical data parsing and initial plotting were performed using base R 4.0.3 functions and GGPLOT2.<br>Ingenuity pathway analysis: Ingenuity Pathway analysis (IPA, Ingenuity® Systems, www.ingenuity.com).<br>Graphpad Prism 8.4.3 (la Jolla, CA, USA).<br>FlowJo software version 10.8.0<br>Proteomic data: Proteome Discover (ver2.5. Thermo Scientific, Waltham, MA, USA).

For manuscripts utilizing custom algorithms or software that are central to the research but not yet described in published literature, software must be made available to editors and reviewers. We strongly encourage code deposition in a community repository (e.g. GitHub). See the Nature Portfolio guidelines for submitting code & software for further information.

Data

Policy information about availability of data

All manuscripts must include a data availability statement. This statement should provide the following information, where applicable:

- Accession codes, unique identifiers, or web links for publicly available datasets
- A description of any restrictions on data availability
- For clinical datasets or third party data, please ensure that the statement adheres to our policy

Raw and analyzed ATAC-seq, RNA-seq, and scRNA-seq data generated in this study are available through the Gene Expression Omnibus under the GEO accession numbers GSE177055 (https://www.ncbi.nlm.nih.gov/geo/query/acc.cgi?acc=GSE177055), GSE181508 (https://www.ncbi.nlm.nih.gov/geo/query/acc.cgi?acc=GSE181508) and GSE187333 (https://www.ncbi.nlm.nih.gov/geo/query/acc.cgi?acc=GSE187333), respectively. The mass spectrometry proteomics data have been deposited to the ProteomeXchange Consortium via the PRIDE partner repository with the dataset identifier PXD033847 and 10.6019/PXD033847. Source data are provided with this paper.

Field-specific reporting

Please select the one below that is the best fit for your research. If you are not sure, read the appropriate sections before making your selection.

☒ Life sciences ☐ Behavioural & social sciences ☐ Ecological, evolutionary & environmental sciences

For a reference copy of the document with all sections, see nature.com/documents/nr-reporting-summary-flat.pdf

Life sciences study design

All studies must disclose on these points even when the disclosure is negative.

Sample size	sample size for in vivo experiments was selected based on power analysis of prior experiments of a similar nature, with an alpha of 0.05, a beta of 0.2 and considering 80% of survival rate after administration of bleomycin.
Data exclusions	No data were excluded from the experiments.
Replication	The bleomycin experiments performed with WT and ERG CKO mice were repeated two times, with different cohorts and with consistent data. Figure legends indicate the number of mice (for in vivo experiments) and the number of biologically independent replicates (for in vitro experiments).
Randomization	For the in vivo studies, no formal randomization method was employed as the genotype of the mice was known before starting the experiment. However, mice were not grouped based on treatment or genotype, but they were randomly distributed in different cages for the entire duration of the experiment.
Blinding	Investigators were not blinded to group allocation as they had to be aware of the groups during separation and treatment steps. Blinding was not used when analyzing results from ATAC-seq, RNA-seq and scRNA-seq due to the unbiased nature of the bioinformatics approaches used to quantify differential expression between age and treatment.

Reporting for specific materials, systems and methods

We require information from authors about some types of materials, experimental systems and methods used in many studies. Here, indicate whether each material, system or method listed is relevant to your study. If you are not sure if a list item applies to your research, read the appropriate section before selecting a response.

Materials & experimental systems

n/a	Involved in the study	
☐	☒	Antibodies
☐	☒	Eukaryotic cell lines
☒	☐	Palaeontology and archaeology
☐	☒	Animals and other organisms
☒	☐	Human research participants
☒	☐	Clinical data
☒	☐	Dual use research of concern

Methods

n/a	Involved in the study	
☒	☐	ChIP-seq
☐	☒	Flow cytometry
☒	☐	MRI-based neuroimaging

Antibodies

Antibodies used	FACS<br>anti-CD45:PerCp-Cy5.5 (103132, clone 30-F11, Biolegend, San Diego, CA, USA, 1:200 dilution), anti-CD31:PE (102407, clone 390,

Biolegend, San Diego, CA, USA, 1:200 dilution), anti-EpCAM:APC (11821335907 , clone G8.8, Biolegend, San Diego, CA, USA, 1:200 dilution), anti CD11b:BV421 (101235, clone M1/70, Biolegend, San Diego, CA, USA, 1:200 dilution), anti Ly6G:FITC (127606, Biolegend, San Diego, CA, USA, 1:200 dilution), anti-Ly6G:BV605 (128035, clone 1A8, Biolegend, San Diego, CA, USA, 1:200 dilution), anti-NK1.1:PEcy5 (108715, clone PK136, Biolegend, San Diego, CA, USA 61-5321-82, Thermo Fisher Scientific, Waltham, MA, USA, 1:3200 dilution), anti-SiglecF:APC-cy7 (565527, cloneE50-2440, BD Biosciences, San Jose, CA, USA, 1:100 dilution), anti-F4/80:APC (123115, clone BM8, Biolegend, San Diego, CA, USA, 1:100 dilution), anti-CD4:APC (1010411, clone GK1.5, Biolegend, San Diego, CA, USA, 1:100 dilution), anti-CD19:BUV395 (563557, clone 1D3, BD Biosciences, San Jose, CA, USA, 1:200 dilution), anti-B220:PE (55309063557, clone RA3-6B2, BD Biosciences, San Jose, CA, USA, 1:200 dilution).

Immunohistochemistry
anti CD31 rat primary antibody (550274, clone MEC 13.3, BD Biosciences, San Jose, CA, USA, 1:200 dilution), anti ERG rabbit primary antibody (97249, Cell Signaling Technology, Danvers, MA, USA, 1:100 dilution).

Immunofluorescence staining
anti CD31 rat primary antibody (550274, clone MEC 13.3, BD Biosciences, San Jose, CA, USA, 1:500 dilution), anti-Collagen IV rabbit primary antibody (NB120-6586, Novus Biologicals, Centennial, CO, USA, 1:200 dilution), anti-Laminin 4 goat primary antibody (NBP2-42392, clone CL3183, Novus Biologicals, Centennial, CO, USA, 1:200 dilution) , anti ERG rabbit primary antibody (97249, Cell Signaling Technology, Danvers, MA, USA, 1:500 dilution), anti alpha-SMA mouse primary antibody (F3777, clone 1A4, Sigma-Aldrich, St. Louis, MA, USA, 1:200 dilution) or anti VE-Cadherin rabbit primary antibody (D87F2, Cell Signaling Technology, Danvers, MA, USA, 1:400 dilution), or anti MPO goat primary antibody (AF3667, R&D System, Minneapolis, MN, USA, 1:200 dilution).

ECM deposition assay
anti-Collagen I (NB600-408, Novus Biologicals, Littleton, CO, USA, 1:200 dilution), anti-ibronectin (sc-9068, Santa Cruz Biotechnology, Santa Cruz, CA, USA, 1:200 dilution).

Validation

All antibodies were commercially available and were validated by the respective manufactures. Additional information can be found at the following links:

Biolegend: https://www.biolegend.com/en-us/bio-bits/highly-specific-validated-antibodies
Antibody validation is a critical step in the journey towards obtaining consistent reproducibility in science. To ensure they are both specific and sensitive, we validate our antibodies through a variety of methods including:
Testing on multiple cell and tissue types with a variety of known expression levels.
Validation in multiple applications as a cross-check for specificity and to provide additional clarity for researchers.
Comparison to existing antibody clones.
Using cell treatments to modulate target expression, such as phosphatase treatment to ensure phospho-antibody specificity.

Novus: https://www.novusbio.com/reproducibility.html
Novus recognizes the need for highly validated, high quality antibodies in the life sciences community. The research community faces ongoing concerns about data reproducibility and especially the validity of antibody-based assays. A recent article in Nature discusses the variable standards and performance of antibodies and antibody suppliers in the market. Novus is committed to addressing this problem and to helping our customers attain the best possible results with our products.
To that end, we actively seek high quality, highly validated products and provide support to ensure that our customers have the tools to properly validate their own assays. We are also collaborating with several global initiatives that help life science researchers choose antibodies with proven results. Of the five pillars of validation established by these initiatives, genetic knockout validation provides the most reliable control for assessing antibody specificity. For technical support in validating your antibody based applications, visit technical support.

R&D systems: https://www.rndsystems.com/quality/antibodies-built-for-reproducibility
With the recent reports stating antibodies as one of the reasons for scientific irreproducibility, you can rest assured with our antibodies. R&D Systems® takes rigorous steps towards antibody validation and reproducibility. We have been since the beginning. For 30 years, we have used our industry-leading production standards and quality control specifications to develop antibodies that can be relied on for specificity and reproducibility. By developing and testing our products in-house, we can ensure a validated and specific antibody. We are confident in our antibodies and provide 100% guarantee for our products. With R&D Systems® antibodies your results will stand the test of time.

Cell signaling Technology: https://www.cellsignal.com/about-us/cst-antibody-validation-principles
At Cell Signaling Technology (CST), we understand that there is no single assay that can determine the validity of an antibody. Confirming that an immunoreagent is sufficiently specific and sensitive depends on the application and protocol being used, the type and quality of sample being analyzed, and the inherent biophysical properties of the antibody itself.
To ensure our antibodies will work in your experiment, we adhere to the Hallmarks of Antibody Validation™, six complementary strategies that can be used to determine the functionality, specificity, and sensitivity of an antibody in any given assay. CST adapted the work by Uhlen, et. al., ("A Proposal for Validation of Antibodies." Nature Methods (2016)) to build the Hallmarks of Antibody Validation, based on our decades of experience as an antibody manufacturer and our dedication to reproducible science.

Millipore/Sigma: https://www.sigmaaldrich.com/US/en/technical-documents/technical-article/protein-biology/elisa/antibody-standard-validation
The success of any immunodetection experiment depends on the quality of the antibodies which are employed. However, antibody reagents vary significantly and when selecting an antibody for a downstream application it is a good idea to spend some time ensuring that not only has it been tested in the chosen experimental setup but that it also demonstrates the required specificity, sensitivity and reproducibility. This valuable data is generated during the antibody manufacturing and validation process and can be found on the product datasheet with which the antibody is supplied.

Abcam: https://www.abcam.com/primary-antibodies/how-we-validate-our-antibodies
To achieve accurate and precise results, you need antibodies that consistently bind specifically and selectively to the intended target. Antibody validation must be application-specific to be effective and information on which applications an antibody has been validated in can be found in the Tested Applications section on any antibody datasheet.
Here you will find more information on what is carried out during our application-specific validation processes.

Eukaryotic cell lines

Policy information about cell lines

Cell line source(s)	human lung microvascular endothelial cells (HLMECs) and human lung fibroblasts (HLF) were purchased from Lonza,
Authentication	cell lines used were not authenticated
Mycoplasma contamination	cell lines tested negative
Commonly misidentified lines (See ICLAC register)	No commonly misidentified lines were used

Animals and other organisms

Policy information about studies involving animals; ARRIVE guidelines recommended for reporting animal research

Laboratory animals	Two and eighteen months old Col1a1-GFP transgenic mice (mixed sex) were kindly provided by Dr. Derek Radisky. Conditional Cdh5-CreER(T)-Ergfl/fl mice (mixed sex) on a C57/BL6 background were generated by breeding of Cdh5-CreER(T) mice with Ergfl/fl mice (Jackson laboratory)
Wild animals	no wild animals were used
Field-collected samples	no field-collected samples were used
Ethics oversight	All animal experiments were carried out under protocols approved by the Mayo Clinic Institutional Animal Care and Use Committee (IACUC) and by the Boston University IACUC and conforming to the ARRIVE guidelines

Note that full information on the approval of the study protocol must also be provided in the manuscript.

Flow Cytometry

Plots

Confirm that:

- [x] The axis labels state the marker and fluorochrome used (e.g. CD4-FITC).
- [x] The axis scales are clearly visible. Include numbers along axes only for bottom left plot of group (a 'group' is an analysis of identical markers).
- [x] All plots are contour plots with outliers or pseudocolor plots.
- [x] A numerical value for number of cells or percentage (with statistics) is provided.

Methodology

Sample preparation	As described in Methods, lungs were minced with a razor blade in a 100 mm petri dish in cold DMEM medium containing 0.2 mg/ml Liberase DL and 100 U/ml DNase I (Roche, Indianapolis, IN, USA). The mixture was transferred into 15 ml tubes and incubated at 37 °C for 35 min in a water bath under continuous rotation to allow enzymatic digestion. Digestion was inactivated with DMEM medium containing 10% fetal bovine serum, the cell suspension was passed through a 40 µm cell strainer (Fisher, Waltham, MA, USA) to remove debris. Cells were then centrifuged (1,350 rpm, 10 min, 4 °C), and resuspended in 3 ml red blood cell lysis buffer (Biolegend, San Diego, CA, USA) for 90 seconds to remove the remaining red blood cells and diluted in 9mL PBS after incubation. Cells were then centrifuged (1,350 rpm, 10 min, 4 °C) and resuspended in 0.2 ml of FACS buffer (1% BSA, 0.5 mM EDTA pH 7.4 in PBS).
Instrument	BD Aria II was used for sorting, BD LSR II SORP was used for FACS analysis
Software	Data were analyzed in Flowjo 10.8.0
Cell population abundance	No tests were performed to asses the purity of each population after sorting

Gating strategy	1) debris exclusion (FSC-A by SSC-A); 2) doublet exclusion (SSC-W by SSC-H and FSC-W by FSC-H); 3) dead cell exclusion (DAPI by CD45).<br>For sorting: CD45 positive cell exclusion (PerCP-Cy5.5 by GFP), EpCAM and GFP positive cells exclusion (APC by GFP), and isolation of CD31 positive cells (APC by CD31).<br>For analysis: Neutrophils (CD45+, CD11b+, Ly6G+), NK cells (CD45+, NK1.1+, Ly6G+), Monocytes (CD45+, CD11b+, Ly6C+), Macrophages (CD45+, SiglecF+, F4/80+), Eosinophils (CD45+, CD11b+, SiglecF+), B cells (CD45+, CD19+, B220+), CD4+ T cells (CD45+, CD4+), CD8+ T cells (CD45+, CD8+).

☒ Tick this box to confirm that a figure exemplifying the gating strategy is provided in the Supplementary Information.
